# Supplementary material for: Patterns of Intron Gain and Loss in Fungi
Source: PLoS Biol. 2004 Nov 30;2(12):e422. doi: 10.1371/journal.pbio.0020422 (PMC532390; doi:10.1371/journal.pbio.0020422)
Supplement: Table S1 — Also available at http://genes.mit.edu/NielsenEtAl/. (4.3 MB ZIP). [file pbio.0020422.st001.zip › NielsenEtAl/html/829.html]

AN6700.1.NCU07922.1.MG09432.1.FG04181.1


```
 CLUSTAL W (1.82) Multiple Sequence Alignments - Introns Inserted


Sequence 1: NCU07922.1	1056 aa
Sequence 2: MG09432.1	1010 aa
Sequence 3: FG04181.1	1045 aa
Sequence 4: AN6700.1	810 aa
Alignment Length: 1065 aa
Number Identitical Residues: 495 aa
Alignment Score (without introns) 26493


MG09432.1 	MSSKENAQSLKVLDELMQKLTISKEADAIKESSNALATFLNGRIEDCDTPTK2TIENLKK
NCU07922.1	MSAKENAQSLKVLDELMQKLTIANEADAVKQASIEVASFINGRTQDLDTPVK2TVEALKK
FG04181.1 	-MPTENQQSIKVLDELFQKLTVSKESSDIKESSNELASFINGRIGDQVVPDN2VIEGLKK
AN6700.1  	----------------------------------------------------~-------
          	                                                            

MG09432.1 	QLGNKKDAAVREKALCAIQAIAQHSNVSPHVEPFLVVLLPSVFAAAGDKITSVKNAALAA
NCU07922.1	QLANKKDATAREKALSAIQAIAQHSEVSAHVEPYLVALLPSVFTAAGDKITAVKNAAIAA
FG04181.1 	QLANKKDATAREKACVAIEAIASHAEVSAAVEPYLVVLLPAVLAAVGDKITAVKNAAQGA
AN6700.1  	------------------------------------------------------------
          	                                                            

MG09432.1 	ASAIAEAINPNAVKATLPHLIESLQNAQKWPEKIAVLDFIETLVRTAPAQTALRVPDLIP
NCU07922.1	ALAIAEAINPNAVKATLNPLIDTIRNAQKWPEKMTALDFIDTLIRTAPVQVGLLVPDLIP
FG04181.1 	VLAIAGGINANAVKAALPCVMESIRSAQKWPEKMAALDFVEYLVKNSPAQLAYRVPELIP
AN6700.1  	------------------------------------------------------------
          	                                                            

MG09432.1 	VVGDAMWDTKKEVKEHAYKIMEKICSLIVNRDIERFIPELIKCIAKPENVPETVHLLGAT
NCU07922.1	VISEAMWDTKKEVKERAYQTMEKLCQLIVNRDIERFIPELIKCIAKPENVPETVHLLGAT
FG04181.1 	VISESMWDTKKEVKERAYKTMEQLCQLIVNKDIERFIPELIKCIAKPENVPETVHLLGAT
AN6700.1  	------------------------------------------------------------
          	                                                            

MG09432.1 	TFVTEVTEPTLALMVPLLERGLQ-ERETAIKRKAAVIVDNMCKLVDDPNIVAAFLPRLMP
NCU07922.1	TFVTEVTEPTLALMVPLLDRGLN-ERDTAIKRKAAVIVDNMCKLVDDPNIVAPFLPKMMP
FG04181.1 	TFVTEVQEPTLALMVPLLDRGLA-ERDTAIKRKTAVIVDNMCKLVDDPNVVAPFLPKMMP
AN6700.1  	-------------MTPLLERGLKAEQATPIKRKAAVIVDNMCKLVEDPQIVAPFLPLLLP
          	             *.***:*** :*: *.****:***********:**::**.*** ::*

MG09432.1 	NLQKN-------FEN-MADPEAREKTKQGLDTLTRVGNVVDGKIPERRNDGDVATVAAHV
NCU07922.1	ALQKN-------YDN-LADPEAREKTKQALDTLVRVGNVVDGKIPEAQNPGDINIVLGHL
FG04181.1 	GLQKN-------YEN-LADPEARDKTKQALDTLTRVGDIKNGVIPEPTFPGAVNVIQPKV
AN6700.1  	QILKLSGESPDPKENGLADPEARGKCKQALDTLTRVGNVVDGKIPEISTAGDISTVSAIL
          	 : * :..:... :*.:****** * **.****.***:: :* ***    * :  :   :

MG09432.1 	RDALSSEKDAKAEKFAPVIEYIAAVGGQLVDEKEVDAPAWATALKPYVSVIVGDAKADSV
NCU07922.1	KEVLGSKYASAFEKSTAIVEYIAAVAGQLVSEKATESSIWAEAVKPYLTVIVGDAESEAV
FG04181.1 	TAALSPKFANYVEKMGPVIEYISAIAGQLVDEKEVESMIWVDNLKAYVSVISGIDNAESI
AN6700.1  	KDILAAKFKSQAEKAEAVINYVAAIAGQLVDEKIAESADWTRNVLPYIAAIVGEADAPAI
          	   *..:     **  .:::*::*:.****.** .::  *.  : .*::.* *  .: ::

MG09432.1 	VETLRKKASPGLADAEEEEADDEEGEDLCNCT2S--------------------------
NCU07922.1	TDALRKKATPGAAEADEAEADDEEGEDLCNCT~FSLAYGAKILLNQTHLRLKRGQRYGLC
FG04181.1 	VENIRKAALPGAVAEAEAEEDEEEGEDLCNCT~FSLAYGAKILLNQTHLRLKRGQRYGLC
AN6700.1  	AETLRKRASPDAAAADAVESDEEEGEDLCNCT~FSLAYGAKILLNQTSLRLKRGQRYGLL
          	.: :** * *. .     * *:**********  : : .:.   ..:    . ..  .  

MG09432.1 	-------------------EGFPKQSEVKTVFVEHDLDSADTEMTTIDWTMKKLEEAKVS
NCU07922.1	GPNGSGKSTLMRAINNEQVEGFPKQSEVKTVFVEHDLDSEDTEMTTIDWTMKKLKEAGVD
FG04181.1 	GPNGSGKSTLMRAINNEQVEGFPKQDEVKTVFVEHDLDSADTEMTTIDWTMKKLEEAGVT
AN6700.1  	GPNGSGKTTLMRAINNEQLEGFPKKDEVKTVYVEHDLDSADTEQTVIGWTMKKLREVGLD
          	....:..::   : .... *****:.*****:******* *** *.*.******.*. : 

MG09432.1 	VTREDVVKRLNEFGFTDSMVSGEITALSGGWKMKLALCRAVFEAPDILLLDEPTNHLDVK
NCU07922.1	TSREDVEAKLAEFGFSPDMIKNDISALSGGWKMKLALCRAVFEAPDILLLDEPTNHLDVK
FG04181.1 	TTQADVEKQLNEFGFTEQMVKGEISALSGGWKMKLALCRAVFEAPDILLLDEPTNHLDVK
AN6700.1  	PVQSEVESKLEEFGFLREQFEGPITALSGGWKMKLALCRAVFEDPDILLLDEPTNHLDVK
          	  : :*  :* ****  . ... *:****************** ****************

MG09432.1 	NVKWLEEYLQNSPCTSIIVSHDSSFLDNVCQHIVHYERFKLKRYRGNLKEFVKKCPSAKS
NCU07922.1	NVKWLEDYLKSSPCTSIIVSHDSGFLDNVCQHIIHYERFKLKRYRGNLKDFVARVPSAKS
FG04181.1 	NVKWLEEYLKNSACTSIIVSHDSGFLDNVCQHIVHYERFKLKRYKGNLAAFVARNPSAKS
AN6700.1  	NVAWLENYLRNSPCTSIIVSHDSKFLDNVLQHVIHYERFKLKRYRGTLSEFVKRVPSARS
          	** ***:**:.*.********** ***** **::**********:*.*  ** : ***:*

MG09432.1 	YYELGASEMEFTFPEPGFLEGVKTKAKAILRATKMDFQYPGTSKPQISDITFQCSLGSRI
NCU07922.1	YYELGASEMEFSFPEPGFLEGVKTKAKAILRATKMSFQYPGTAKPQISDISFQCSLGSRI
FG04181.1 	YYELGESEIEFSFPEPGFLEGVKTKAKAILRATNMSFQYPGTSKPQISDISFQCSLGSRI
AN6700.1  	YYELGASDLEFKFPEPGFLEGVKTKAKAIIRVSNMYFRYPGVDRDQLQDITFQVSLGSRI
          	***** *::**.*****************:*.::* *:***. : *:.**:** ******

MG09432.1 	AVIGPNGAGKSTLINVLTGELIPTAGEIYQHENIRIAYIKQHAFAHIDNHLDSTPSEYIQ
NCU07922.1	AVIGPNGAGKSTLINVLTGELIPTQGEIYQHENIRIAYIKQHAFAHIDNHLDKTPSEYIQ
FG04181.1 	AVIGPNGAGKSTLINVLTGELIPTQGEIYQHENIRIAYIKQHAFAHIDNHLDKTPSEYIQ
AN6700.1  	AVIGPNGAGKSTLVDVLTGEKIATRGEVYHHENIRIAYIKQHAFAHIDNHLDKTPSEYIQ
          	*************::***** *.* **:*:**********************.*******

MG09432.1 	WRFQTGEDRETMDRANKIITEEDEKAMDKVFKVDGTQRRVIGINARRKFKNSYEYECTFA
NCU07922.1	WRFQTGEDRETMDRANKIITEADEEAMNKVFKIEGTPRRVIGIHSRRKFKNSYEYECSFA
FG04181.1 	WRFQTGEDRETMDRANKIITEADEKAMDKVFRIEGSQRRVIGINSRRKFKNSYEYECSFA
AN6700.1  	WRFQTGEDRETMDRANKQVTEEDEKAELKIYKIEGTPRRVVGVHARRKFKNTYEYECSFL
          	***************** :** **:*  *:::::*: ***:*:::******:*****:* 

MG09432.1 	LGENIGMKNERWVPMMSADNAWLPRSELLASHQKMVAEVDMKEALASGQFRPLVRKEIES
NCU07922.1	LGENIGMKNERWVPMMTADNAWLPRNELLASHQKMVADVDMKEALASGQFRPLVRKEIES
FG04181.1 	LGENVGMKNERWVPMMSADNAWLPRNELLASHQKMVADVDMKEALASGQFRPLVRKEIES
AN6700.1  	LGENIGMKSEKWTPMSTTDNAWIPRNEIMESHSKKVAEIDAQEALKSGNIRPLIRKEIED
          	****:***.*:*.** ::****:**.*:: **.* **::* :*** **::***:*****.

MG09432.1 	HCANFGLDAELVSHSRMRGLSGGQRVKVVLAACSWQRPHLIVLDEPTNYLDRDSLGALSK
NCU07922.1	HCANFGLDAELVSHSRMRGLSGGQRVKVVLAACSWQRPHLIVLDEPTNYLDRDSLGALSK
FG04181.1 	HCANFGLDAELVSHSRMRGLSGGQRVKTVLAACSWQRPHLIVLDEPTNYLDRDSLGALSK
AN6700.1  	HCAQFGLDAELVSHSQMRGLSGGQRVKVVLAACSWQRPHVIVLDEPTNYLDRDSLGALSK
          	***:***********:***********.***********:********************

MG09432.1 	ALKKFEGGVIIITHSSEFTKDLTEEVWAVVDGKMTPSGHNWVQGQGSGPRLKADDGEEEE
NCU07922.1	ALKKFEGGVIIITHSAEFTKDLTEEVWAVMDGKMTPSGHNWVQGQGSGPRLKEDDGEEEE
FG04181.1 	ALKKFEGGVIIITHSAEFTKDLTEEVWAVMDGKMTPSGHNWVQGQGAGPRLKADDGDEEE
AN6700.1  	ALKAFEGGVIIITHSAEFTQNLTEEVWAVLDGKMTPSGHNWVTGQGSGPRLEEKEG-PDE
          	*** ***********:***::********:************ ***:****: .:*  :*

MG09432.1 	KFDAMGNKIVSTKKAKKLTSAEARK~KKKERMARRKRGEE~VFSDEDE-
NCU07922.1	KFDAMGNKIVSTKKKAKLSSAELRK~KKKERMARRKRGEE0VFSDEDDF
FG04181.1 	KFDAMGNKIVTTKK----------K0KKKERMARRKRGEE~VFSDEDDL
AN6700.1  	IVDAMGNKIKVEKK-VKLSSGDKRK~QRKERLARKKRGED~VDDDEDF-
          	 .*******   **  . ::..  * ::***:**:****: * .***
```
